# Clashes of consensus: on the problem of both justifying abortion of fetuses with Down syndrome and rejecting infanticide

**DOI:** 10.1007/s11017-017-9398-8

**Published:** 2017-02-10

**Authors:** Henrik Friberg-Fernros

**Affiliations:** Department of Political Science, University of Gothenburg, Gothenburg, Sweden

**Keywords:** Abortion, Infanticide, Down syndrome, Criteria of consciousness, Viability, Good Samaritan argument

## Abstract

Although the abortion of fetuses with Down syndrome has become commonplace, infanticide is still widely rejected. Generally, there are three ways of justifying the differentiation between abortion and infanticide: by referring to the differences between the moral status of the fetus versus the infant, by referring to the differences of the moral status of the act of abortion versus the act of infanticide, or by separating the way the permissibility of abortion is justified from the way the impermissibility of infanticide is justified. My argument is that none of these ways justifies the abortion of fetuses diagnosed with Down syndrome while simultaneously rejecting infanticide. Either the justification for abortion is consistent with infanticide, or it is implausible to justify abortion while rejecting infanticide. I conclude the article by making some preliminary remarks about how one might manage the situation posed by my argument.

## Introduction

I begin with the following thought experiment. Suppose it were impossible to diagnose fetuses with Down syndrome, such information being obtainable only after birth. Would it then be justifiable to kill infants diagnosed with Down syndrome? I believe that most of us intuitively would not consider such killing justifiable.^1^ People with Down syndrome often apparently have a good quality of life, making the euthanasia of infants with Down syndrome difficult to justify.^2^ Killing infants who have Down syndrome for other reasons seems even more difficult to justify. Nevertheless, screening fetuses for Down syndrome has become a common obstetric practice in many countries [5], and as no treatment is available for the defects detected, the screening is intended to provide information that can serve as a basis for deciding whether or not to abort the fetus [6]. The exact termination rate is uncertain, though it is clear that a great majority of fetuses diagnosed with Down syndrome are aborted.^3^ Considering how “routinized” screening for Down syndrome has become, at least in the Western world, and how high the termination rate is when this defect is detected, I believe it is reasonable to conclude that we, in practice, have an emerging consensus about the permissibility of aborting fetuses with Down syndrome—although this does not mean that the practice is uncontroversial.

Despite the widespread acceptance of such abortion, most people still overwhelmingly reject the infanticide of those with Down syndrome (if that diagnosis is made only after birth), raising the question of how to reconcile these beliefs. I provide reasons for doubting that one can do so, specifically defending the claim that the criteria invoked to distinguish between abortion and infanticide are either unable to justify why aborting fetuses with Down syndrome is permissible while infanticide is not or are too implausible to justify the abortion of fetuses diagnosed with Down syndrome.

This article is structured as follows. First, I will demonstrate why the argument—specifically with respect to consciousness and viability—advanced to justify aborting fetuses with Down syndrome would also justify infanticide, and I will counter some objections to this conclusion. I will then examine two other attempts to justify aborting fetuses with Down syndrome while rejecting the permissibility of infanticide: appeal to the significance of birth and the argument of bodily integrity (the “Good Samaritan argument”). I criticize both attempts by demonstrating that they have highly implausible implications. I then turn to a third strategy for justifying abortion while rejecting infanticide. The two abovementioned strategies aim to identity a common ground for justifying abortion rights and differentiating abortion and infanticide. For example, fetal viability is used to justify abortion while, at the same time, justifying the rejection of infanticide. In contrast, the third strategy for justifying abortion while rejecting infanticide is to find one way of justifying abortion, and—if it turns out that this criterion cannot exclude the permissibility of infanticide—another way of rejecting infanticide. I discuss these attempts in the third section of the article, and defend my claim that these also fail for the same reasons as the other two ways failed: either these ways are compatible with infanticide or they are implausible. I end the article by summarizing my argument and considering different options for how we might act if we grant that my arguments are sound.

## The problem of differentiating between abortion of fetuses with Down syndrome and infanticide

Perhaps the most obvious way of arguing that abortion is permissible while infanticide is not is by considering the differences between fetuses and infants. However, because fetuses with Down syndrome are aborted quite late in pregnancy,^4^ the differences are limited. Nevertheless, several have been proposed in the literature and I think one can categorize them as follows: first, criteria related to the existence of consciousness (e.g., desires and sentience), the criterion of viability, and the criterion of birth.^5^ According to these criteria, fetuses aborted due to Down syndrome lack consciousness and, therefore, also desires and sentience; they would not survive outside the mother’s womb and have not yet been born. While these differences constitute potential grounds for justifying abortion while rejecting infanticide, I will argue that all but the last criterion—that of birth—are unsuccessful because they are inconsistent with a principled rejection of the moral permissibility of infanticide. In contrast, though the criterion of birth is indeed consistent with the principled rejection of infanticide, it should be rejected as well because it is highly implausible, which I hope to demonstrate in the third section below.

I will start by considering criteria related to the existence of consciousness and then turn to the criterion of viability in order to demonstrate why these criteria are compatible with the moral permissibility of infanticide. The criteria related to consciousness do not exclude infanticide simply because an infant can be born without having been conscious at earlier stages. In fact, Professor Hugo Lagercrantz concludes that, generally, “extremely preterm infants born before 25 weeks are probably not conscious at birth”—which of course supports my claim, although it is sufficient to demonstrate that an infant “can” be born without having been conscious at a previous stage [14, p. 304]. Moreover, Lagercrantz states that these preterm infants do not “wake up and show signs of consciousness” [14, p. 304]. The possibility of infants being born without having been conscious means that they also can be born without having been sentient or having had desires.

One might try to defend criteria based on the existence of consciousness while still holding that infanticide is wrong by arguing that one cannot know for sure that the fetus lacks consciousness before birth. This is in fact how David Boonin defends his criterion based on organized cortical activity. Boonin concludes that conscious desires, which he maintains are the basis of a right to life, “occur at some point from 25 to 32 weeks after fertilization”; he nevertheless proposes that adopting a more conservative position “seems advisable given our lack of definitive knowledge” [15, p. 128]. By adopting such a conservative position, which would rule out the possibility that preterm-born fetuses could survive outside the womb, abortion could be justified while infanticide is rejected. Abortion would then be morally permitted up to 20 weeks of gestation and thereafter be impermissible due to the mere risk of fetuses exhibiting some kind of consciousness.

Some support for this position is provided in the medical literature. Here is how two scientists put it, when commenting on the emergence of fetal consciousness:

If we are to accept that by approximately 20 weeks the requisite neural substrate of consciousness (e.g., the thalamus and associated subcortical structures) and its proper connections are in place and accompanied by a coordinating EEG rhythm (even if only intermittently), what can we say about the beginning moments of fetal consciousness? Again, it would seem that we can conclude that consciousness is at least possible from this point forward in fetal development. [16, p. 87]

These scientists do not rule out the possibility that consciousness might emerge only after 20 weeks of gestation, a position that might be considered in line with Boonin’s conservative position. However, it is one thing to justify the mere possibility of consciousness but quite another to justify the position that this mere possibility should be ascribed such moral importance as to constitute a right to life, which in turn would determine the moral permissibility of infanticide. Consequently, not only is consciousness at this fetal stage empirically uncertain, it is also uncertain what ethical relevance such consciousness should then be ascribed (see [17, 18]).

In this regard, one must distinguish between at least two kinds of consciousness:

The first is “consciousness as the waking state” and the second is “consciousness as experience.” Consciousness in the first sense is the behavioral expression of the waking state. Being conscious in that sense is synonymous to being alert and awake. The second sense of consciousness, however, refers to becoming aware of something and to experiencing something, which is often called “phenomenal consciousness.” [17, p. 88]

As the thalamocortical connections must be established before fetuses can be conscious in the latter sense, and as this happens no earlier than after 25 gestational weeks, Boonin and other proponents of consciousness-based criteria must justify why the mere possibility of consciousness in the former sense constitutes a right to life. So far that has not been done; indeed, the fact that Boonin invokes organized cortical activity as the criterion for when the fetus can be ascribed a right to life implies that he rejects such a position.

However, for the sake of argument, I set aside this objection and assume that one can justify the position that the mere possibility of some kind of consciousness constitutes a right to life as early as in week 20. Such a position would still not rule out the moral permissibility of infanticide according to criteria based on the existence of consciousness. This is so because one can eliminate this uncertainty about whether or not fetuses in week 20 are conscious by artificially suppressing the emergence of fetal wakefulness altogether.^6^ For example, one could anaesthetize the fetus and thereby prevent it from waking up at all.^7^ It would then be permissible to kill the infant once it has been delivered according to criteria based on the existence of consciousness.^8^ Such a procedure would certainly be feasible using current medical technology.

Admittedly, my argument here suggests that we gain certainty about the lack of fetal consciousness only by *artificial* means, which would pave the way for justifying infanticide. Does not this fact undermine the argument? I do not think so: the fact that the emergence of consciousness is prevented by artificial means is not, as I see it, decisive. Many ethical dilemmas arise due to our use of new technology. For example, it is only because it is possible to save extremely preterm fetuses using advanced medical technology that the question arises about whether the infanticide of fetuses without cortical cortexes is permissible.

A similar objection would question the permissibility of anaesthetizing fetuses by drawing on the distinctions between allowing and doing or the principle of double effect. By anaesthetizing the fetus in order to be able to kill the infant, you actively and intentionally pursue an action which might eventually result in harm for the infant (who might be killed). Would that not be impermissible? Certainly, that might be true, though not according to consciousness-based criteria. The fetus lacks a right to life as long as it lacks consciousness, which is why abortion is permissible according to such criteria up to, at least, week 20. If it is permissible to actively and intentionally eliminate the fetus by abortion up to week 20 according to these criteria, then it is arguably also permissible to anaesthetize it actively and intentionally since the latter causes less harm than the former. Therefore, this objection to my argument against invoking precautionary concerns in order to differentiate between abortion before week 20 and the infanticide of extremely preterm fetuses without a functioning cortical cortex also fails.

Even if it were both feasible and permissible to render a fetus unconscious by artificial means once it has been born in order to be permitted to kill it, what would be the point? Why would a woman choose to deliver an anaesthetized fetus? To establish that such an action would be rational is unnecessary for defending my main thesis in this section, namely, that consciousness-based criteria are compatible with infanticide. Nevertheless, it would strengthen my case if one could demonstrate that it would be rational under some circumstances to act in such a way, and I believe there are such circumstances. For example, if some birth defects cannot be detected when the fetus is in the womb, or if such detection is highly risky when the fetus is in the womb, then it might be rational to keep the fetus unconscious until delivery in order to be permitted to choose whether or not to kill the infant.

That the parents should have such a right has recently been defended by Alberto Giubilini and Francesca Minerva [23], labeling it, oxymoronically, “after-birth abortion.” According to them, the fact that some pathologies are likely to remain undetected until delivery makes it urgent to address the question of whether it is morally permissible to kill infants born with such pathologies. Especially relevant to my argument is how they present their case for the after-birth abortion of infants with Down syndrome:

An examination of 18 European registries reveals that between 2005 and 2009 only 64% of Down syndrome cases were diagnosed through prenatal testing. This percentage indicates that, considering only the European areas under examination, about 1700 infants were born with Down syndrome without parents being aware of it before birth. Once these children are born, there is no choice for the parents but to keep the child, which sometimes is exactly what they would not have done if the disease had been diagnosed before birth. [23, p. 261]

Giubilini and Minerva do not accept the consciousness-based criteria for differentiating between abortion and infanticide; rather, they argue that infanticide should be accepted if abortion is, as a matter of consistency. Their article illustrates, however, that there are intelligible claims for permitting infanticide because some defects are not detectable during pregnancy. By anaesthetizing the fetus, such an action would be permissible under criteria based on the existence of consciousness.

Viability is another criterion that can be invoked to defend abortion while rejecting infanticide. Viability as a criterion of fetal status means that the fetus is able to survive outside the womb. According to the criterion in this version, it is permissible to abort a pre-viable fetus because it is only after viability that the fetus is considered a person entitled to a moral right to life. Historically, fetal viability has tended to occur increasingly early in the pregnancy as an effect of technological developments. At present, it is possible for fetuses to survive outside the womb after 22–23 weeks of gestation. The inability of the fetus to survive outside the womb makes abortion permissible, according to this version of the viability criterion, while still holding that infanticide is impermissible given that it would involve the killing of a viable infant.

One common objection to this version of the viability criterion is that it is implausible that a human being’s possession of rights should be dependent on the development of technology. As medical technology develops, fetuses become viable earlier in pregnancy, implying that the basic rights of the human being have successively changed during the course of history. Given this implication, it seems reasonable to ask, rhetorically, as William Cooney does, “[can] personhood be a condition relative to and dependent on technology?” [24, p. 161]. There have been a few attempts to defend this criterion, but as many commentators have noted, implausible implications seem inevitably to undermine it. For example, according to this criterion, a conjoined twin whose survival is dependent on being connected to the other twin would not have full moral status as a human [25, p. 51; 26, p. 25; 27, p. 438]. I believe that such a conclusion is a *reductio ad absurdum* argument against this criterion.

However, for the purpose of this article, it is unnecessary to accept this conclusion, because applying the criterion of viability in the case of conjoined twins illustrates a more trivial and less controversial conclusion: the criterion of viability cannot rule out the permissibility of killing a conjoined twin who is dependent on the other twin. Indeed, as the twin whose survival is dependent on being connected to the other twin is not viable, it has no right to life and can be killed on the same grounds on which an unviable fetus can be aborted. Consequently, neither viability criterion nor the other consciousness-based criteria can simultaneously justify both the permissibility of abortion and the impermissibility of infanticide.^9^

So far, I have demonstrated that consciousness-based criteria and the criterion of viability are compatible with infanticide, which means that one cannot reject infanticide while holding that abortion is permissible based on these criteria. Admittedly, the circumstances in which infanticide is permissible according to these criteria are rare. This is particularly the case when it comes to viability; according to this criterion, only conjoined twins dependent on the other twin can permissibly be killed. Still, the mere fact that these criteria are compatible with infanticide under certain rare circumstances undermines the position that aborting fetuses with Down syndrome is permissible and infanticide impermissible. That is so because, from the point of view of these criteria, there is no ethically relevant difference between an extremely preterm infant without a functioning cortical cortex and a conjoined infant dependent on the other twin, on one hand, and a fetus just diagnosed with Down syndrome, on the other. Abortion criteria based on consciousness or viability cannot consequently rule out the permissibility of infanticide.

## Why the criterion of birth and the argument from bodily integrity cannot justify the abortion of fetuses with Down syndrome

So far, I have argued against reconciling the beliefs that aborting fetuses with Down syndrome is permissible while infanticide is not by demonstrating that the criteria invoked to justify the abortion of fetuses with Down syndrome—those based on consciousness or viability—are, in fact, consistent with the permissibility of infanticide. In other words, one cannot use these criteria to differentiate between abortion and infanticide because they permit both actions. However, two other criteria are able to differentiate between abortion and infanticide, namely, the criterion of birth and the argument of bodily integrity (or the Good Samaritan argument). Still, as I hope to demonstrate here, neither criterion can justify the abortion of fetuses with Down syndrome due to its implausible implications. I will start by discussing the criterion of birth, followed by the Good Samaritan argument.

When the criterion of birth is applied, abortion of fetuses with Down syndrome and infanticide are differentiated by referring to the fact that the infant has been born while the fetus is still in the womb. This criterion is also problematic, however, because differentiating between the fetus and the infant based solely on where they are located seems unjustifiable.^10^ Robert Wennerberg nicely summarizes this criticism: “Surely personhood and the right to life is not a matter of location. It should be *what* you are, not *where* you are that determines whether you have a right to life” [29, p. 98] (emphasis in the original). Second, suppose that the infant being outside the womb is sufficient to indicate that infanticide is impermissible. The following thought experiment can be conducted. There is just enough of a life-saving substance to save either an embryo in vitro or a fetus just about to be born, but not both. We would then be obliged, according to this view, to give the substance to the embryo rather than to the fetus because the embryo is located outside the womb. That would indeed be implausible, not because it would be implausible to save the embryo, but because it would be implausible to choose the embryo over the fetus just about to be born simply because of the former’s location. This implication demonstrates the implausibility of this criterion and why it should be rejected as a criterion for abortion in general.^11^

A way to avoid this implication is to say that being outside the womb is not a sufficient but only a necessary criterion for being entitled to a right to life. The entity outside the womb must also meet other criteria in order to be entitled to a right to life. However, such a defense undercuts the justification based on the differentiation between a fetus and an infant due to location, as other criteria must be considered to determine the impermissibility of infanticide. If these other criteria are absent, then the criterion of birth is insufficient to justify the permissibility of abortion while rejecting the permissibility of infanticide. Consequently, the criterion of birth cannot then justify why it is permissible to abort fetuses with Down syndrome but impermissible to conduct infanticide.

Faced with the failure to justify the differentiation between abortion and infanticide by referring to differences between the moral status of the fetus and the infant, one might turn to the moral difference between the act of abortion and the act of infanticide. According to this argument, there is no difference between the moral status of the fetus and the infant; on the contrary, both entities are assumed to have a right to life. Instead, it is the fact that the fetus, unlike the infant, is dependent on the woman’s life-sustaining assistance that potentially provides a justification for aborting fetuses with Down syndrome while rejecting infanticide. It is argued that, as the woman has no obligation to maintain her life-sustaining treatment, it is permissible to terminate the pregnancy by abortion without violating the rights of the fetus (as long as it is non-viable), while such an action is not available after birth. This line of argument is sometimes called the good Samaritan argument (hereafter, GS argument) for the permissibility of abortion, as it claims that requiring the woman to refrain from abortion would be like requiring her to act as a good Samaritan, which is an unjustifiable demand.

Proponents of the GS argument claim that abortion is permissible even if the fetus is assumed to be a person. This position is defended by the use of a well-known analogy about a violinist who depends on another in order to survive. The philosopher Judith Jarvis Thompson applied this analogy to the GS argument when it was introduced in 1971. Here is how it goes:

You wake up in the morning and find yourself back to back in bed with an unconscious violinist. A famous unconscious violinist. He has been found to have a fatal kidney ailment, and the Society of Music Lovers has canvassed all the available medical records and found that you alone have the right blood type to help. They have therefore kidnapped you, and last night the violinist’s circulatory system was plugged into yours, so that your kidneys can be used to extract poisons from his blood as well as your own. The director of the hospital now tells you, “Look, we’re sorry the Society of Music Lovers did this to you—we would never have permitted it if we had known. But still, they did it, and the violinist is now plugged into you. To unplug you would be to kill him. But never mind, it’s only for nine months. By then he will have recovered from his ailment, and can safely be unplugged from you.” [31, pp. 48–49]

Thomson argues that it would be permissible for you to unplug yourself from the violinist even though this act would lead to the death of the violinist. Similarly, Thompson thinks that a woman has the right to abort a fetus even though one assumes, for the sake of argument, that it would lead to the death of another person since the aim would be to avoid the burden of pregnancy rather than to kill the fetus. Moreover, as this reason cannot be invoked in order to justify infanticide, this argument claims to be able to differentiate between abortion and infanticide.^12^

This defense of the permissibility of abortion is very controversial. One of its most prominent defenders, David Boonin, concludes that even though many believe it to be ingenious, most still consider it flawed.^13^ However, to make my argument against the permissibility of aborting fetuses with Down syndrome as strong as possible, I will disregard such criticism and merely assume that the violinist case is sufficiently analogous to a pregnancy and demonstrate that, even so, the abortion of fetuses with Down syndrome would still be impermissible.

Down syndrome is detectable by tests that can be conducted as soon as the end of the first or the beginning of the second trimester [34]. This implies that information about the fetus having Down syndrome is always preceded by information about the pregnancy and that one can therefore assume that it is not the information about the pregnancy that leads to the decision to abort, but rather, the later information about Down syndrome. Moreover, the fact that a fetus has Down syndrome does not in itself generate an extra burden during pregnancy; rather, the extra burden is expected to occur after birth.^14^

To test whether an abortion under these circumstances would be permissible, I must adjust Thomson’s thought experiment about the violinist. Given that the information about the fetus having Down syndrome is preceded by the information about the pregnancy, I assume that an individual decides to maintain her life-supporting assistance when she realizes that she is connected to the violinist, but that she changes her mind after being informed about the status of the violinist. To be as analogous as possible to the abortion of fetuses with Down syndrome, I assume that the reason she decided to stay connected to the violinist in the first place was that she expected the future existence of the violinist to be more beneficial than burdensome to herself. However, when the violinist is examined three months after she is first connected, it is suggested that the future existence of the violinist would burden her more than it would benefit her. She changes her mind and she disconnects herself.

Now, the decisive question is whether it would be permissible for the individual to disconnect herself from the violinist once she realizes that his future existence will be burdensome to her. The burden of the pregnancy is not the only reason she unplugs herself since she seems to have initially accepted—at least temporarily—the arrangement, as she did not disconnect herself from him until the examination of the violinist three months later. The aim of avoiding the burden of being connected is not consequently sufficient for the decision to disconnect herself; without the information about the violinist being a future burden to her, the disconnection would not have taken place. What does that say about the intention of the disconnection in that circumstance?

It could be that the individual aims to eliminate the existence of the violinist simply because he would be a burden to her if he survived. In that case, a disconnection would obviously be morally impermissible. To illustrate this point, one might assume that she actually enjoyed being connected to the violinist, but once she realized that the violinist would be a burden to her in the future, she decided to disconnect herself in order to eliminate the existence of the violinist. The only aim of her action would consequently be to secure the death of the violinist. If the intention of an action is morally relevant, then surely such an intention makes the disconnection morally impermissible. Rather than being an unfortunate side effect, the death of the violinist is then an intentional effect. This distinction is also endorsed by proponents of Thomson’s argument as they usually emphasize that the mother’s right to terminate life-sustaining treatment is not a right to “kill the fetus per se” but rather the right “to decide she does not want to use her body to sustain the fetus’s life” [33, p. 334] (see also [13; 15, p. 219]). And indeed, as I show below, proponents of the GS argument need to adhere to this view—according to which the moral status of an action is affected by the intention—in order for their argument to work.

More realistically, however, the individual’s aim in disconnecting after realizing that the violinist may become a burden to her is not solely to eliminate the existence of the violinist but also to avoid the burden of being connected for a couple of months. For sure, avoiding these inconveniences was not a sufficient reason for disconnection. As long as she did not know that the violinist would be a burden to her in the future, she agreed to stay connected, but once she realized that he would be a burden, she concluded that it was not worth staying connected to him. Is that morally impermissible as well?

I believe so. As long as the intention to secure the death of the violinist is an essential element of the decision to disconnect oneself, I believe that the decision would be impermissible. Drawing on Boonin [15, p. 218], an essential element of an action can be identified by answering the following counterfactual question: would the individual have disconnected herself if she then would secure the death of the violinist? And the answer here is obviously “no” since she agreed to stay connected as long as she remained unaware about the fact that the violinist would be a burden to her. That demonstrates how essential the intention to bring the morally bad outcome in terms of securing the death of the violist is for her decision to unplug herself; securing the death of the violinist is therefore also an intentional act rather than a side effect of her act to disconnect in this case. Granting that this case is sufficiently analogous to the situation in which a fetus has been diagnosed with Down syndrome—which, for instance, means that it is assumed that the fetus has a right to life—and given that it is impermissible to intentionally secure the death of the violinist, the conclusion follows that abortion in that case would be impermissible.

This conclusion, however, clearly depends on the distinction between intended and foreseen effects of an action defended by the principle of double effect. Another way to object to my conclusion above—and to defend abortion of fetuses with Down syndrome—is to reject the moral relevance of this distinction. Does the intention of the action really matter as long as the action is the same? I believe that this distinction is well founded, but it is neither feasible nor necessary for my present purposes to defend this position since it is obvious that the GS argument itself is dependent on the justification of that distinction in order to differentiate between abortion and infanticide. This is so because if it were permissible to abort a fetus diagnosed with Down syndrome merely in order to ensure the death of the fetus—as it would be in the first case, where the individual chooses abortion despite the fact that she actually enjoined being pregnant—then it seems hard to defend the position that it would be necessarily impermissible to kill an infant diagnosed with Down syndrome.

In both cases, the proponents of the GS argument assume that human persons are being killed. Moreover, while the pregnant woman actually enjoined being pregnant, the parents who realize that their infant has Down syndrome experience the situation as being very burdensome. Why would it be morally permissible in that situation to abort the fetus, but not to kill the infant? I do not see that the GS argument can provide any answer to that question; the mere fact that one human person is located within a womb while another person is outside the womb does not seem sufficiently plausible. Rather it is the difference with regard to the moral status of the actions that address the burden of (unwanted) pregnancies and of (unwanted) infants respectively that generally justifies the differentiation between abortion and infanticide, and that difference is due to the intentions of the acts. Therefore, proponents of the GS argument must differentiate between the moral status of abortion and infanticide by invoking the distinction between actions with foreseen effects (like avoiding the burden of being pregnant) and intentional effects (like infanticide). However, this argument implies that abortion with the intention of ensuring the death of the fetus—as the one I referred to above—is impermissible, which means that abortion of fetuses with Down syndrome is generally not permissible as they involve an intention to ensure the death of the fetus.

Consequently, the GS argument cannot be invoked to justify the permissibility of aborting fetuses with Down syndrome. This is not primarily because this argument is unable to differentiate between the abortion of fetuses with Down syndrome and the killing of infants with Down syndrome, as is the case, as I have argued, with criteria based on consciousness and viability. On the contrary, the most plausible version of the GS argument can differentiate between the abortion of fetuses with Down syndrome and infanticide. The reason why the GS argument cannot be used to justify the abortion of fetuses with Down syndrome is rather that such an action would be impermissible under the premises of its own argument.

## Differentiating abortion and infanticide by external criteria

So far, I have focused on different ways of trying to justify abortion that do not lead to the conclusion that infanticide might also be permissible. This strategy, if it had worked, would have been the most robust way of defending abortion rights while, at the same time, rejecting the permissibility of infanticide. It would have provided a way to both justify abortion rights while differentiating between abortion and infanticide. To illustrate, with consciousness, the fetus’s *lack* of consciousness explains why abortion is permissible while *the existence* of consciousness of the infant explains why infanticide is impermissible. My claim is, however, that this attempt fails, as do the other ways under discussion here. Either the criteria justify both abortion and infanticide (as is the case with the criteria of consciousness and viability) or they are simply too implausible to justify abortion of fetuses with Down syndrome (as is the case with the criteria of birth and the GS argument).

Faced with this result, another strategy may be to abandon the aim of finding a way to simultaneously justify both abortion and its differentiation from infanticide. Instead, one might try to settle on the most plausible way of justifying abortion, and if that justification leads to the conclusion that infanticide is also permissible under certain circumstances, then seek other *external* parameters to differentiate infanticide from abortion. By external parameters, I mean parameters that are not related to the justification for abortion.^15^ For example, one might hold on to the criterion of consciousness as a way of justifying abortion while accepting the claim that this criterion does not rule out infanticide. Then, in order to rule out infanticide, one might instead invoke other differences between unconscious fetuses and infants that justify a differentiation between abortion and infanticide. One such difference that has previously been invoked is the possibility of giving up an infant for adoption [35, p. 20; 36, p. 29; 37]. If there are persons ready to adopt the child once it is born, then it is possible to avoid the burden of being a parent without killing the infant. In contrast, a fetus as such cannot be adopted before it is born, which means that there is a difference between abortion and infanticide with regard to adoption. There are, of course, other differences, but I will start by discussing adoption, and then make some general claims about this strategy to justify a differentiation between abortion and infanticide by invoking external parameters.

One immediate response to this line of thought is to claim that, if it is granted that adoption is available, adoption is not only an alternative to infanticide but also a potential alternative to abortion. Admittedly, adoption can only be implemented after delivery, but this mere difference in timeline does not invalidate adoption as an alternative to abortion, granted that adoption is considered to be an alternative to infanticide. It would be more reasonable to suggest that it is the burden of being forced to give birth before adoption that differentiates abortion and infanticide. Since adoption as an alternative to abortion requires that the baby is born, while adoption as an alternative to infanticide does not, this difference might justify why abortion is permissible while infanticide is not. Consequently, it is the burden of being forced to give birth to the baby before she or he can be given up for adoption that justifies abortion but not infanticide.

The differences with regard to the magnitude of burden that the implementation of adoption requires does not, however, seem to provide a general justification for differentiating abortion from infanticide. Firstly, it depends on whether the baby can be given up for adoption—if no one is ready to adopt the child (and no other way exists to avoid parenthood once the child is born), then, of course, there would be no difference between abortion and infanticide in this regard. Secondly, it is conceivable that allowing abortion but not the infanticide of infants with Down syndrome imposes a greater burden than allowing infanticide while not allowing abortion of fetuses with Down syndrome—despite the fact that adoption might be viewed as a less costly alternative to infanticide than to abortion.

To illustrate this, imagine a situation in which abortion, infanticide, as well as adoption are allowed. Consequently, in order to avoid becoming a parent to a child with Down syndrome, one can eliminate the fetus by abortion, kill the infant, or give the infant up for adoption. The question is whether it would be *necessarily* more burdensome to outlaw abortion rather than infanticide merely because adoption is a less costly alternative to infanticide than abortion. The proponents of this argument need to justify an affirmative answer to this question in order to defend the differentiation between abortion and infanticide, and I do not think that they can accomplish this.

Even if most people were to consider outlawing abortion to be more burdensome than outlawing infanticide, it is not implausible to believe that some will think otherwise. Remember that those seeking to abort a fetus with Down syndrome have initially accepted the burden of pregnancy—given that they decide to abort the fetus only after it is found to have Down syndrome. Consequently, some might think that the pregnancy itself is not the primary problem and therefore not very burdensome—even though this category would probably constitute a minority since it seems reasonable to assume that most women would consider it to be burdensome to give birth to a child merely to give it up for adoption. Nonetheless, for some, the burden of pregnancy might be quite manageable; their central aim, rather, may be to avoid becoming a parent to a child with Down syndrome by extinguishing the offspring.^16^ Granted that this is the aim, adoption is not an alternative to either infanticide or abortion, which in turn means that adoption cannot provide the general justificatory basis for differentiating abortion from infanticide.

Moreover, as I mentioned above, there may be reasons—as the philosophers Giubilini and Minerva have claimed—to opt for infanticide rather than abortion as the latter implies a certain risk that a healthy fetus is eliminated while this risk can be ruled out in case of infanticide [23]. Therefore, if the aim is to avoid becoming a parent of a child with Down syndrome by extinguishing it, infanticide is safer than abortion. Now, one can certainly question the legitimacy of choosing to kill the infant rather than to give it up for adoption—and I will do that later on—but that is not at issue here.^17^ Rather, my aim has been to demonstrate that it is not necessarily more burdensome to outlaw abortion rather than infanticide merely because adoption generally is a less costly alternative to the latter compared to the former since adoption might be irrelevant as an alternative. Therefore, the criterion of adoption cannot generally justify why abortion is permissible while infanticide is not.

There are, of course, other external differences between abortion and infanticide situations that can be invoked to justify a differentiation between these two actions. For example, in the latter situation, there is commonly both a father and a mother equally affected by the situation, while the mother is clearly more affected in the former situation [35]. However, this difference—like others of an external nature, I dare to claim—does not categorically rule out the permissibility of infanticide since it is a contingent difference. Consequently, if, for example, egg donations are permitted by anonymous donors, there may be cases where only the mother is left to decide whether or not the infant should be killed, which, in turn, eliminates this difference between the cases and makes infanticide permissible according to this criterion.

Once one fails to find a criterion that justifies abortion in a way that rules out the permissibility of infanticide, it seems difficult to identify other parameters that could justify a general rejection of infanticide. Recall that some of the previously discussed parameters are too implausible—i.e., the criterion of birth and the GS argument—and there are, of course, other differences that are even more obviously implausible. For example, an infant can be observed with the naked eye while the fetus can only be observed by ultrasound, but to invoke this difference as a justification for why infanticide is impermissible but abortion is not seems highly implausible. Other parameters fail because they are contingent and therefore can be arranged in way that eliminates the difference between abortion and infanticide, which, in turn, invalidates the justification for the differentiation. In either case, one fails to justify abortion while rejecting the permissibility of infanticide.

## Concluding remarks

The overall conclusion of the present arguments is that it is difficult to morally justify the abortion of fetuses with Down syndrome without also permitting the killing of infants with Down syndrome. There are at least three kinds of associated difficulties. First, I believe that I have demonstrated that criteria based on the existence of consciousness and the criterion of viability are compatible with infanticide. Second, the remaining criteria for differentiating between infanticide and abortion—the criterion of birth and the GS argument—are not sufficiently plausible to justify the abortion of fetuses with Down syndrome. Third, it seems difficult to identify external criteria—i.e., parameters that are not related to the justification of abortion—that are sufficiently plausible or cannot be arranged to eliminate the relevant differences between abortion and infanticide situations. My conclusion, therefore, is that it seems problematic to both justify abortion of fetuses with Down syndrome and, at the same time, to reject the permissibility of infanticide.

If this conclusion is correct, and if we care about how we justify our actions, what approach should be taken toward abortion and infanticide? One option is to hold on to consciousness-based criteria or the viability criterion to justify the abortion of fetuses with Down syndrome, and to accept infanticide under circumstances in which these criteria allow it. However, if so, the implications must also be recognized, that is, fetuses can permissibly be anaesthetized in order to prevent consciousness and then killed after they have been born and a conjoined twin—dependent on his or her twin to survive—can permissibly be killed. Another option is to reject these criteria and not accept the permissibility of infanticide under these circumstances. But if my claim that the other ways of justifying the abortion of fetuses with Down syndrome—namely, criterion of birth and the GS argument—fail, then it follows that it is morally impermissible to abort fetuses with Down syndrome. A third option is to accept consciousness-based criteria or viability as a way to justify the abortion of fetuses with Down syndrome and then try to find other, external criteria to differentiate between fetuses with Down syndrome and unconscious or non-viable infants. However, as I have tried to demonstrate, it might be hard to find external criteria that are able to provide a general justification for the differentiation between fetuses with Down syndrome and unconscious or non-viable infants. Which of these three positions are the most reasonable?

While I will not be able to accomplish a full-fledged defense of my position here, I will nevertheless provide reasons for why I believe that we should opt for the second position. I have not argued against abortion as such in this article, nor have I provided reasons against infanticide. Nonetheless, I believe it is plausible to conclude that it is at least uncertain whether it would be morally right to permit infanticide or whether it would be morally disastrous to do so. In support of this conclusion, one can invoke arguments in favor of the position that infants generally have a right to life, which makes it at least plausible to fear that infanticide would violate that right to life of the infant. Now, if infanticide were allowed, and it turned out that infanticide actually violated a human person’s right to life, then we would commit a gravely wrong action. Such a scenario speaks in favor of the second option. One could, moreover, invoke the fact that it would not be very costly to avoid making infanticide permissible. As discussed above, adoption provides an alternative to infanticide, and as long as parenthood can be avoided by means other than killing the fetus, such an option seems preferable to infanticide. Admittedly, the cost of not permitting women to abort fetuses diagnosed with Down syndrome would generally be higher—even if it cannot be ruled out, as I demonstrated above, that it would be less costly than infanticide in some circumstances. Nonetheless, as I mentioned above, adoption is also an option in these cases [39]. Equally, there are plausible arguments in favor of the view that fetuses with Down syndrome also have a right to life, which means that permitting their abortion might be gravely wrong. Therefore, in the face of these uncertainties, there are strong reasons to opt for the second alternative whereby both the abortion of fetuses with Down syndrome and infanticide are rejected.

## Footnotes

1 I will take this position for granted, even though few polls confirm this conviction; for one exception, see [1].

2 The literature reports that the quality of life of people diagnosed with Down syndrome can be quite high; see, for example, [2–4].

3 An early review of relevant published studies suggests a termination rate of 92% [7], while a more recent review considering only studies of the termination rate in the USA concluded that the rate was 67–85% [8].

4 According to Hume and Chasen [9], the median age at prenatal diagnosis in the US (2012-2014) was 12 weeks.

5 This selection, which is based on my review of the literature, excludes certain alternative criteria. Two currently fairly unpopular such views are “quickening” (see, for example, [10]) and delayed hominization (see, for example, [11]). As these views are less often defended than those discussed above, I offer no full refutation of them, but I believe that some of the arguments against the consciousness-based criteria are relevant also in relation to them (see below). One might also object that the gradualist position should be included in my selection. According to this position, the moral status of the fetus increases over the course of pregnancy (see, for example, [12]). However, the gradualist position does not by itself define when abortion is impermissible and is therefore not offering any specific distinction between fetuses and infants that potentially could justify aborting fetuses with Down syndrome while rejecting infanticide (cf. [13]). My claim is thus that proponents of the gradualist position are, if they want to justify aborting fetuses with Down syndrome, ultimately restricted to choosing among specific criteria of which those selected here are the most important.

6 It is important also to note that uncertainties about whether the fetus can experience sensations or desires are eliminated if the fetus is anesthetized. Although recent research suggests that it is possible to have “perceptions without awareness,” that would still require that the one perceiving not be asleep in a way that an anesthetized fetus would be (cf. [19]). Rather, anesthetization is known to prevent sensations such as pain. Moreover, although it is likewise possible to have an “unconscious desire,” for example, to live while one is asleep, that does not apply to fetuses anesthetized early in pregnancy because a fetus cannot have an unconscious desire unless it has once had some kind of desire (cf. [15, p. 126]). By anesthetizing the fetus before the capacity for having desires arises, the possibility of unconscious desires is consequently eliminated.

7 As the fetus can be the subject of medical interventions such as open surgery, which might generate fetal pain, medical procedures are available to anaesthetize the fetus (see [20, 21]).

8 I would argue that proponents of quickening and delayed hominization theory are also vulnerable to this objection—granted that they offer criteria that aim to justify the abortion of fetuses with Down syndrome. Like accounts based on consciousness, the criterion of quickening as well as of delayed hominization that are invoked to justify the abortion of fetuses with Down syndrome seem to depend on the assumption that the fetus is awake. Most naturally, a fetus which is not awake does not move in a way that is necessary for “quickening.” Therefore the criterion of quickening is dependent on the assumption that the fetus is awake. And since this criterion does not provide a justification for why it would be impermissible to prevent wakefulness through, for example, anesthetization of the fetus, an account based on the criterion of quickening is vulnerable to the same objection as consciousness-based criteria. In an admittedly less obvious way, an account based on a criterion of delayed hominization that aims to justify abortion of fetuses with Down syndrome also seems vulnerable to this objection. According to this theory, fetuses are ensouled once there is a biological capacity to receive the soul. This usually means that the brain must be sufficiently developed in order to produce rudimentary manifestation of a rational soul with intellect and will. As one of the leading contemporary proponents of this view puts it: “having a soul of certain sort requires having the actual powers associated with that soul…” [11, p. 529]. But a fetus that is not awake cannot have “the actual powers”—for example “higher level thoughts”—which are associated with a rational soul. Neither can a fetus which is not awake be spiritually active—which is claimed to be another condition for ensoulment according this account [22, p. 83]. Consequently it seems like anesthetization of the fetus—by which the fetus is prevented from being wakeful—would simply be another obstacle for the ensoulment of the fetus, just as an undeveloped brain constitutes an obstacle for ensoulment according to this view. Accordingly, an account based on delayed hominization is also vulnerable to the objection above.

9 This conclusion is certainly relevant even to cases in which fetuses do not have Down syndrome. As long as the fetus has not been diagnosed with injuries severe enough that killing it after birth could potentially be justified from the point of view of their best interest, this conclusion is valid. However, given that a very small percentage of abortions is performed late in pregnancy, and given that fetuses with Down syndrome are seldom diagnosed before week 12, even as a very high percentage of fetuses diagnosed with Down syndrome are aborted, I believe that this category of late abortions is of special interest. I have therefore focused on this kind of abortion even though my conclusions have a wider scope of relevance.

10 See, for example, [23, 27]. One can certainly claim that it is not the difference in location per se that matters but, more specifically, the fact that the fetus is located in a woman’s womb while the infant is not (see, for example, [28]). However, by this line of reasoning, it is not birth per se that matters; rather, the defense of abortion with reference to the birth criterion instead collapses into the defense of abortion with reference to bodily rights. I argue against such a defense of the abortion of fetuses with Down syndrome below.

11 For a similar line of argument, see [30, p. 31].

12 For a recent defense of abortion rights based on this line of reasoning, see [32, 33].

13 Indeed, the organization of Boonin’s rigorous defense of this argument in *A Defense of Abortion* suggests that this argument has been criticized in no fewer than 16 ways; see [15, ch. 4].

14 Certainly worries about the future of the child might afflict the future parents during a pregnancy with a Down syndrome diagnosis more than during a pregnancy without this diagnosis. However, such worries are still about the consequences of the diagnosis and the possible physiological sequelae for the parents (and the rest of the family) after birth.

15 This distinction draws on Mary Ann Warren’s distinction between intrinsic and relational properties as a theoretical basis for rights [28]. However, external parameters are a wider category that includes not only relational properties but also other parameters.

16 This view is endorsed by some feminists. For example, the political scientist Sarah Langford emphasizes that the aim of an abortion is sometimes to “prevent the existence of their biological children and thus prevent motherhood” [38, p. 267]. Boonin also acknowledges that this can be an aim with abortion; see [15, p. 221].

17 This could, of course, be questioned, since one might defend the argument for adoption against infanticide by arguing against the legitimacy of aiming to eliminate one’s offspring by abortion. If that could be achieved, then adoption would be a more appropriate alternative to infanticide than abortion (since the latter but not the former can be justified due to the aim of avoiding the burden of pregnancy), which in turn, might justify the differentiation between abortion and infanticide. However, abortion proponents must identify a way that justifies the rejection of the permissibility of the aim to eliminate the infant or the fetus—rather than solely to avoid parenthood—that does not restrict the abortion rights based on either consciousness-based criteria or viability, and that seems hard to achieve. For example, one can, of course, decide not to invoke the right to life of fetus as a reason why the aim to eliminate it would be impermissible, since that would restrict abortion rights. More generally, the problem can be formulated in the following way: once one has accepted that reasons can override the reasons for the permissibility of *infanticide* other than those based on criteria related to consciousness or viability, then it seems, out of consistency, that one also must accept that other reasons can override the reasons for the permissibility of *abortion* based on these criteria. If this conclusion is correct, then this way of defending abortion while rejecting infanticide also is not successful in the end.
